# Effect of Exogenous Addition of Microplastics on the Ability of Plants and Soil to Accumulate Thallium

**DOI:** 10.3390/toxics14030250

**Published:** 2026-03-12

**Authors:** Jinjin Wang, Pengfei Che, Junlie Zhou, Jian Luo, Shunbin Lan, Xiuxiang Meng, Huibin Shi, Jinzhao Hu

**Affiliations:** 1 School of Environment and Resource, Xichang University, Xichang 615013, China; jinjin.wangjj@gmail.com (J.W.);; 2 School of Earth Sciences, Yunnan University, Kunming 650500, China; 3 School of Ecology and Environment, Renmin University of China, Beijing 100872, China; 4 School of Earth System Science, Tianjin University, Tianjin 300072, China; 5 Neijiang Normal University, Neijiang 641100, China

**Keywords:** hyperaccumulator species, geo-accumulation index, potential ecological risk, heavy metal contamination, phytoremediation

## Abstract

Thallium (Tl) contamination of soils in lead-zinc mining areas poses potential ecological risks, and the impact of microplastics on Tl accumulation by hyperaccumulator plants remains unclear. This study examined soils collected from the Daliangzi lead-zinc mining area to investigate the characteristics of Tl contamination. These soil samples were used in plant cultivation experiments. The thallium contents in both the soil and plant samples were determined using acid digestion followed by inductively coupled plasma mass spectrometry (ICP-MS). The contamination level, plant enrichment capacity, and ecological risk were then comprehensively evaluated through the Geo-accumulation index (*I_geo_*), Bioconcentration factor (BCF), and potential ecological risk index. The results indicated that the *I_geo_* of Tl in the soil was 2.413, corresponding to a moderately to heavily polluted level, which necessitates focused attention. Polyethylene exhibited markedly opposing effects on *Pteris vittata* and *Solanum nigrum*: it significantly promoted Tl accumulation in the former, while distinctly inhibiting it in the latter. Microplastics could modify the pH value of soil, as well as the contents of nitrogen (N) and phosphorus (P). Risk assessment indices indicated that Tl pollution in this region reaches a very high contamination level with moderate potential ecological risk. Polyethylene and polypropylene demonstrated a species-specific promoting effect on Tl adsorption by the two hyperaccumulator plants, with polyethylene significantly enhancing the Tl accumulation capacity of *P. vittata* and polypropylene distinctly promoting Tl adsorption in *S. nigrum*.

## 1. Introduction

Microplastics (MPs, <5 mm) and heavy metals (HMs), are two types of environmental pollutants with distinct characteristics; however, they widely coexist. In recent years, this has resulted in persistent, cumulative contamination in the global ecosystem, posing a severe challenge to ecological security and human health [1,2,3,4]. MPs and HMs are widely distributed in various environmental media, e.g., air ecosystems, water environments (seas, beaches, estuaries, rivers, and lakes), and soil [5,6,7,8,9]. As anthropogenic polymeric fragments, MPs demonstrate a strong adsorption affinity for HMs owing to their high specific surface area and abundant surface functional groups. Therefore, MPs can serve as important carriers for the migration and transformation of HMs in the environment [10,11,12,13,14].

Thallium (Tl), which is recognized as a highly toxic HM, can cause severe neurological and organ damage when exposed to the environment [15,16]. Consequently, it is strictly regulated in numerous countries, such as China, the United States, and Canada, which have incorporated it into their environmental priority pollutant lists [17,18,19]. The concentration of Tl in the Earth’s crust and soil is relatively low (approximately 0.3 μg g^−1^ and 0.2 μg g^−1^, respectively) [20]. Thallium mainly exists in sulfide deposits (such as lead-zinc ores), and anthropogenic activities act as the principal source of thallium contamination in the environment, resulting in a significant increase in concentrations across diverse environmental media. For example, reported concentrations are as follows: 1968 μg L^−1^ in groundwater [21], 16.5 μg L^−1^ in mine water [22], 8.18 μg L^−1^ in stream water [23], 2.91 μg L^−1^ in surface water [22], 71.60 μg g^−1^ in soil [24], 4.15 μg g^−1^ in plants [25], and 41 ng m^−3^ in the atmosphere [26]. As a result, enhanced monitoring and regulation of Tl in the environment are imperative to safeguard ecological and human health. The adsorption process of thallium by microplastics is mainly controlled through electrostatic attraction, surface complexation, and hydrophobic interactions. It has been demonstrated that MPs, which typically possess a strong negative surface charge, can adsorb Tl(I) through electrostatic interactions [27]. PS exhibits a strong adsorption capacity for Tl through complexation with its surface oxygen-containing functional groups [20]. Studies by Zhou et al. [28] and Liu et al. [29] demonstrated that polyamide exhibits the highest adsorption capacity for Tl(I) among common plastics such as polystyrene (PS), polyethylene terephthalate (PET), and polyvinyl chloride (PVC).

The adsorption and accumulation mechanisms of plants with respect to MPs and HMs have emerged as a crucial focus in contemporary environmental remediation research [30,31]. This area of investigation not only elucidates how plants adapt to the stress of combined pollution but also provides a scientific basis for the development of green and sustainable ecological remediation technologies. For instance, Chebbi et al. [32] demonstrated that the presence of MPs in contaminated soil from mining areas leads to an increase in the accumulation of HMs in the shoots of alfalfa. Huang et al. [33] discovered that MPs could enhance the absorption of Cd by ryegrass and promote the transfer of Cd from roots to stems and leaves to varying extents. Furthermore, Xu et al. [34] demonstrated that MPs enhance HM uptake in lettuce by enriching the relative abundance of key metal-mobilizing bacteria in the rhizosphere. In conclusion, the absorption of HMs by plants through MPs is an effective way to reduce soil HM pollution. However, this process has dual ecological effects: on the positive side, plants remove and reduce HMs in the soil through MPs; however, from the perspective of risks, the enrichment of HMs on the surface of MPs may alter their environmental fate and more efficiently transmit toxicity into plants and even the food chain. Therefore, future research should not only confirm the remediation potential of this process but also prioritize the quantitative assessment of its long-term effects on plant physiology and ecology, the transformation patterns of HM speciation, and their ultimate fate within ecosystems, so as to ensure the safety and sustainability of its environmental application.

Although co-contamination by HMs and MPs has drawn increasing attention, the influence of microplastics on the thallium accumulation capacity of plants remains unclear, which limits a comprehensive understanding of the environmental behavior of this type of combined pollution. To fill this gap, the soil in a typical lead-zinc mining area was selected as the research object in this study, with the aims of: (1) assessing the pollution and enrichment characteristics of Tl in the soil; (2) revealing the influence of MPs on thallium adsorption by plants; and (3) clarifying the effect of MP addition on the ecological risk of the soil.

## 2. Materials and Methods

### 2.1. Study Areas and Sample Collection

**Figure 1:**
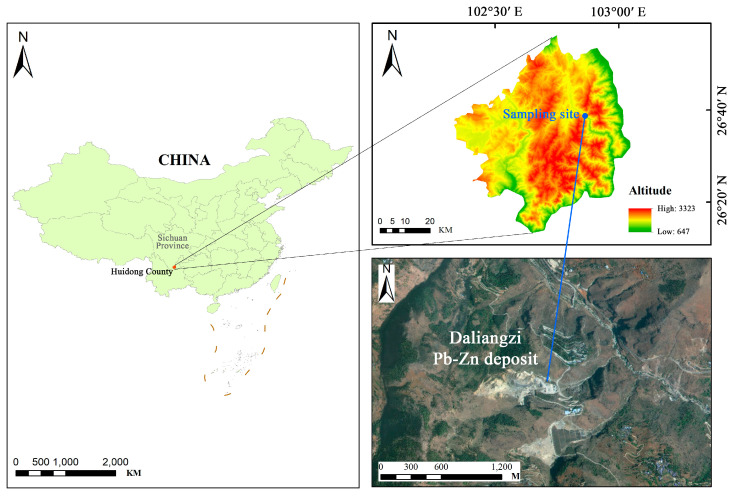
Sampling location map.

The Daliangzi lead-zinc deposit (DLZ) is located in Qianxin Town, Huidong County, Liangshan Yi Autonomous Prefecture (Figure 1). As the second-largest lead-zinc deposit in Sichuan Province, it is characterized by substantial resource reserves estimated at approximately 4.5 Mt. The ore exhibits average grades of 0.75% Pb and 10.5% Zn, with sphalerite being the predominant mineral [35]. Notably, thallium is a significant trace element associated with the deposit; its reported concentration ranges from 0.001 to 10.94 mg kg^−1^ in geological materials from the area, with a mean of 0.58 ± 1.42 mg kg^−1^ [36]. To investigate Tl mobility and contamination in the environment, soil samples were systematically collected from the DLZ mining area (102°52′14″ E, 26°37′41″ N) in July 2023. Using a pre-cleaned stainless-steel shovel, three composite surface soil samples (0–10 cm depth) were obtained, each composed of multiple sub-samples from within a defined sampling plot.

Immediately after collection, the soils were placed in large, labeled, self-sealing polyethylene bags to prevent cross-contamination and moisture loss during transport to the laboratory. Subsequent sample preparation followed rigorous procedures. In the lab, the collected soils were first spread on clean trays and air-dried naturally at ambient temperature to a constant weight. Following drying, visible plant debris and coarse particles were manually removed. The samples were then ground thoroughly using an agate mortar to achieve a homogeneous fine powder and sieved through a 200-mesh (<75 µm) nylon sieve to ensure uniform particle size for subsequent geochemical analyses. This preparation protocol was designed to facilitate accurate and reproducible determination of elemental concentrations, including Tl and associated HMs.

### 2.2. Experimental Study

This study employed a controlled pot experiment to simulate and assess the effects of different types of microplastics on the phytoremediation potential of two locally sourced plant species in soil from a lead-zinc mining area. The experiment began in October 2023 using a pot-culture simulation approach. Two native plant species, namely *Pteris vittata* (*P. vittata*) and *Solanum nigrum* (*S. nigrum*), which are recognized for their metal tolerance and accumulation capabilities, were chosen for the trial. The experimental design comprised five distinct treatment groups. A blank control group (CK) was established with soil containing no added microplastics. The remaining four treatment groups were individually amended with one of four prevalent microplastic polymers: polyethylene (PE), polypropylene (PP), polyvinyl chloride (PVC), or polyethylene terephthalate (PET), each added at an identical concentration.

The soil preparation protocol was conducted as follows: Initially, soil samples collected from the target lead-zinc mining area were air-dried, homogenized, and sieved to remove coarse debris such as gravel, plant residues, and roots. Then the treated soil was evenly filled into pre-cleaned custom-made glass culture pots with a volume of approximately 2.5 L. For the control group (CK), the soil was used without any microplastic addition. For each microplastic treatment, a predetermined mass (0.45%, *w*/*w*) of the respective polymer (PE, PP, PVC, or PET) was thoroughly and uniformly mixed into the soil matrix prior to potting to ensure consistent exposure. Prior to transplanting, uniform healthy seedlings of *P. vittata* and *S. nigrum* were obtained from the standardized nursery of Xichang University. All seedlings were cultivated in an HM and MP-free seedling substrate under consistent nursery conditions, to ensure the uniformity of initial growth status. Seedlings with consistent growth were selected for transplanting: *P. vittata* and *S. nigrum* seedlings featuring 2–3 leaves. Subsequently, the screened seedlings were transplanted into the correspondingly treated soils, with one seedling planted in each pot. After transplanting, a 7-day seedling recovery period was set, and poorly growing seedlings were replaced during this period to ensure the consistency of the initial growth status of the plants in all pots. All pots were maintained under natural light and temperature conditions without artificial climate control. During the formal cultivation period, environmental parameters were continuously monitored: the daily average temperature ranged from 13.9 °C to 27.4 °C; the average daily illumination duration was 10.2–11.5 h. The plants were watered every 2–3 days with filtered ultrapure water to maintain the soil water holding capacity at 60–70% of the field capacity, avoiding waterlogging throughout the experiment. After the 45-day formal cultivation period, both the plant and soil samples were systematically harvested for subsequent analysis of Tl and MP interactions.

Both experimental soil and plant samples were collected in pre-cleaned glass bottles to prevent external contamination. Upon returning to the laboratory, the plant samples were thoroughly rinsed with ultrapure water to remove surface-adhered soil and impurities. Subsequently, they were placed in an oven and heated at 105 °C for 1 h to deactivate enzymes and arrest biological activity. Afterward, they were dried at 65 °C until a constant weight was achieved. All dried plant samples were pulverized into a fine and homogeneous powder using a ball mill (Model LC-PBM, LICHEN, Shaoxing, China). The same drying and grinding procedures were applied to the soil samples to ensure consistency in subsequent analyses.

The digestion of all samples, including the field soil, experimental soil, and plant materials, was conducted in accordance with the assisted acid digestion protocol described by Wang et al. [37]. In brief, precisely 0.1000 g of each powdered sample was accurately weighed and placed into a digestion vessel. Subsequently, 1 mL of HNO_3_ (UP) and 1 mL of HF (UP) were added in sequence. The vessels were sealed and placed into a temperature-controlled digestion system. Digestion was performed under a programmed heating profile: the temperature was held at 100 °C for 1 h, raised to 180 °C and maintained for 29 h, and then lowered to 140 °C for an additional 6 h to ensure the complete dissolution of silicate matrices and organic components. After cooling, the resulting digestates were diluted to a final volume of 10 mL with ultrapure water and filtered prior to analysis. The concentrations of HMs (e.g., Tl, Ba, Cr, V, and Pb) were measured via Inductively Coupled Plasma Mass Spectrometry (ICP-MS; NexION^®^ 2000, PerkinElmer Inc., Waltham, MA, USA). Calibration was carried out using multi-element standard solutions, and quality assurance was ensured by employing certified reference materials, method blanks, and duplicate samples throughout the analytical process.

The pH of the soil samples was determined via potentiometric titration in accordance with the NY/T 1377–2007 standard, using water as the extractant at a soil-to-water ratio of 1:2.5 [38]. Soil available nitrogen and phosphorus were determined using a soil nutrient rapid tester (Model TPY-6A, TP, Ningbo, China).

### 2.3. Statistical Analysis

ArcGIS Pro (Version 3.6, USA) was used to map the sampling locations; Data were statistically analyzed using Excel (2024, USA) and Minitab (Version 20.0, USA); Graph plotting was carried out using Origin (2021, USA).

### 2.4. Assessment Methods

To systematically assess the degree of contamination, characteristics of ecological migration, and potential hazards of Tl in the study area, this study employed multiple environmental geochemical evaluation methods (Table 1). First, the Geo-accumulation Index (*I_geo_*) was used to quantitatively classify and evaluate the extent of anthropogenic Tl contamination in soil or sediments. Second, the Bioconcentration factor (BCF) was used to analyze the transfer capacity of thallium from soil to plants. Finally, the potential ecological risk index (*E_r_*) was used to comprehensively evaluate the toxicity response level and potential ecological hazards posed by Tl.

## 3. Results and Discussion

### 3.1. HM Pollution Levels in Different Types of Soil

**Figure 2:**
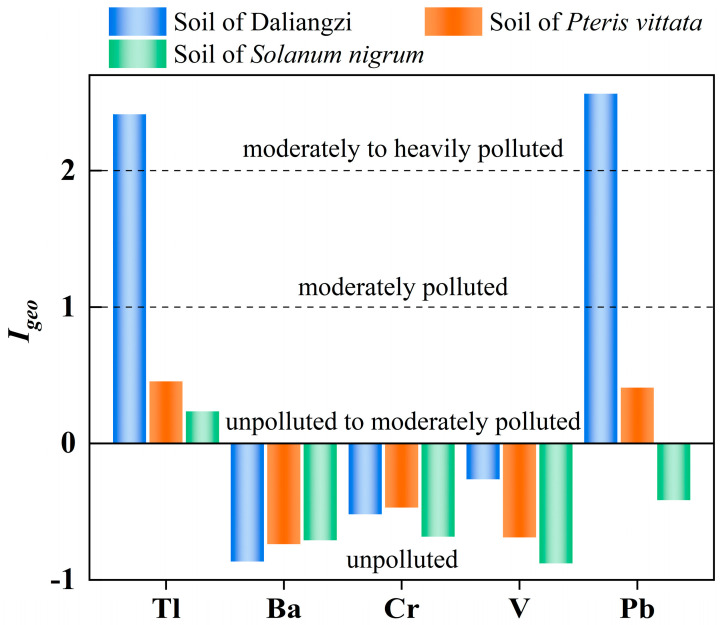
*I_geo_* variation for soil samples of type: Soil of Daliangzi, Soil of *Solanum nigrum* and Soil of *Pteris vittata* for Tl, Ba, Cr, V and Pb and comparison of contamination levels. (Tl: thallium, Ba: barium, Cr: chromium, V: vanadium; Pb: lead).

The geo-accumulation index (*I_geo_*) analysis results for the HMs (Tl, Ba, Cr, V, and Pb) in different soil types are presented in Figure 2. The highest degrees of contamination were observed for thallium and lead, with *I_geo_* values of 2.41 and 2.56, respectively, classifying them as moderately to heavily contaminated. On the other hand, the *I_geo_* values of Ba, Cr, and V were consistently low, falling within the uncontaminated range. It is worth noting that the pollution of Tl and Pb in the soil was effectively alleviated through the implementation of phytoremediation measures. Specifically, *P*. *vittata* reduced the *I_geo_* values of Tl and Pb in the soil to 0.45 and 0.24, respectively, while *S*. *nigrum* reduced them to 0.41 and −0.42, respectively. Li et al. [43] demonstrated that *P. vittata* exhibits strong tolerance to low concentrations of thallium (≤20 μg L^−1^) in wastewater containing Tl, with its accumulation in the plant showing a dynamic translocation pattern from roots to shoots. Furthermore, Wu et al. [44] demonstrated that *S. nigrum* has a strong accumulation capacity for low concentrations (<10 mg kg^−1^) of Tl in soil, with that in its roots being the strongest. This study identified Tl as the primary pollutant in the soil surrounding the Daliangzi Pb-Zn mine and experimentally demonstrated the feasibility and effectiveness of using *P. vittata* and *S. nigrum* for the remediation of this contaminated soil.

### 3.2. Tl Enrichment Coefficient of Plants After the Addition of MPs

**Figure 3:**
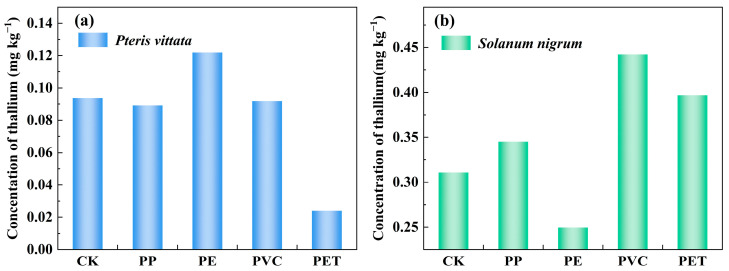
The influence of MP types of PP, PE, PVC and PET on Tl accumulation in plants: (**a**) *Pteris vittata*, (**b**) *Solanum nigrum*. (CK: blank control group; PP: polypropylene; PE: polyethylene; PVC: polyvinyl chloride; PET: polyethylene terephthalate).

**Figure 4:**
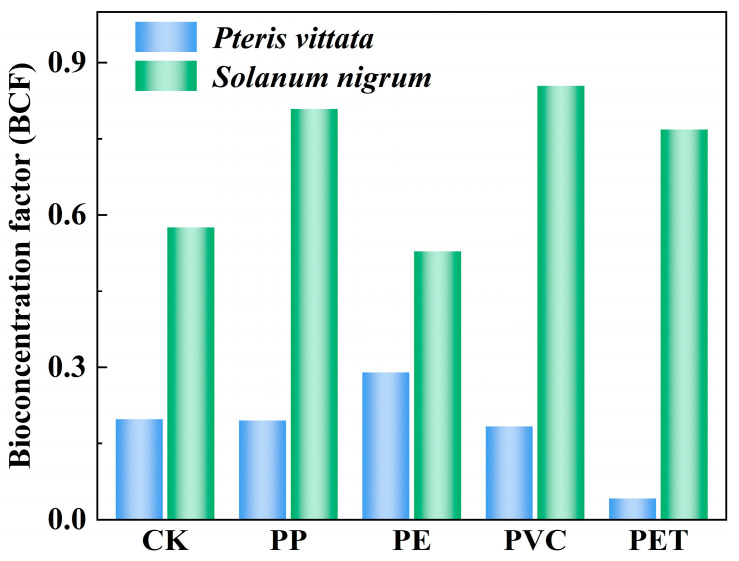
Comparison of the BCF of Tl in plants *Pteris vittata*, and *Solanum nigrum* influenced by MP types: PP, PE, PVC and PET. (CK: blank control group; PP: polypropylene; PE: polyethylene; PVC: polyvinyl chloride; PET: polyethylene terephthalate).

As shown in Figure 3 and Figure 4, the type of MP significantly affected the accumulation of Tl in the two repair plants. Specifically, for *P. vittata*, compared to the CK group (0.094 mg kg^−1^), treatment with PE increased its Tl content to 0.122 mg kg^−1^, indicating a promoting effect. In contrast, treatments with PP, PVC, and PET reduced its Tl content by 0.004 mg kg^−1^, 0.002 mg kg^−1^, and 0.070 mg kg^−1^, respectively (Figure 3a). These three types of MPs inhibited the absorption of Tl by *P. vittata*. Compared to the CK group, the treatments ranked in the following order according to the Tl BCF of *P. vittata*: PE (0.29) > PP (0.20) > PVC (0.18) > PET (0.04). This is similar to the findings reported by Marzi et al. [45], which also indicated a predominance of PE in *P. vittata*. It is widely recognized that *P. vittata* is a typical arsenic hyperaccumulator, and its absorption of HMs predominantly depends on root-specific phosphate transporter proteins [46]. The roots of *P. vittata* can secrete oxalic acid, thereby increasing the solubility of HMs in the rhizosphere [47]. Furthermore, the distinct promotional effect of PE observed in this study may be attributed to its specific physicochemical properties or surface characteristics. These properties and characteristics could interact with the rhizosphere environment and potentially upregulate the expression of Tl-related transporter proteins in *P. vittata*. Beyond direct MP-plant interactions, the role of rhizosphere microorganisms cannot be overlooked. Plant growth-promoting bacteria (PGPB) have been shown to improve phytoremediation efficiency in soils co-contaminated with MPs and HMs by modulating the structure and function of the rhizosphere microbial community. For instance, studies have confirmed that PGPB alleviate Cd + PE stress and improve Cd phytoremediation efficiency [48].

In contrast, *S. nigrum* demonstrated a distinct response pattern to MPs. Compared to the CK group (0.311 mg kg^−1^), PE treatment decreased its Tl content to 0.250 mg kg^−1^, indicating an inhibitory effect. Conversely, treatments with PP, PVC, and PET increased its Tl content by 0.034 mg kg^−1^, 0.131 mg kg^−1^, and 0.086 mg kg^−1^, respectively, demonstrating a promoting effect (Figure 3b). The BCF analysis revealed that the Tl accumulation capacity of *S. nigrum* under different MP treatments ranked in the following order: PVC (0.85) > PP (0.81) > PET (0.77) > PE (0.53). An inhibitory effect of PE on HM accumulation in *S. nigrum* was also observed in a study by Xu et al. [49], which reported that high-dose PE significantly inhibited the growth of *S. nigrum* and reduced both Cd concentration and accumulation in the plants. This polymer-dependent response underscores the necessity of considering the specific types of MPs, rather than MPs as a whole, when assessing their role in plant-HM interactions and designing effective phytoremediation strategies for co-contaminated soils.

### 3.3. The Influence of MPs on the Physical and Chemical Properties of Soil

**Figure 5:**
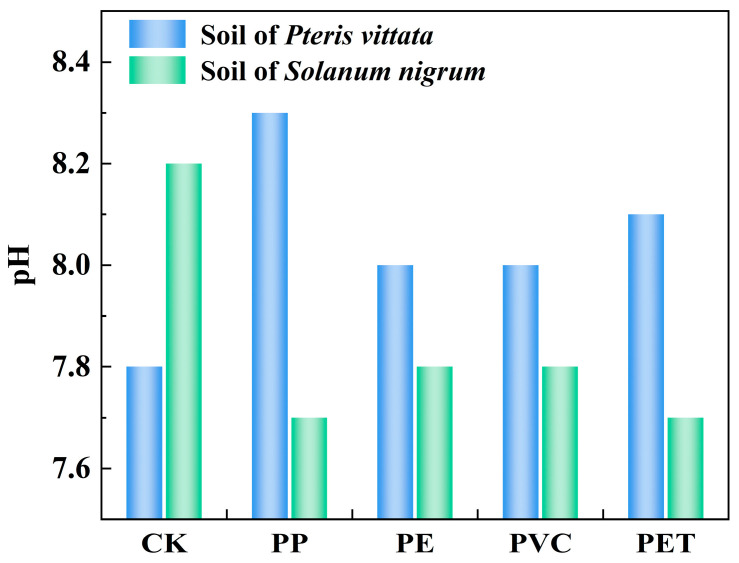
Effects of various MPs on soil pH in the presence of Tl. (CK: blank control group; PP: polypropylene; PE: polyethylene; PVC: polyvinyl chloride; PET: polyethylene terephthalate).

As shown in Figure 5, the effects of different types of MPs on the pH value of Tl-contaminated soil exhibit certain differences. In soils cultivated with *P. vittata*, the addition of MPs led to an increase in pH. Specifically, PP raised the pH by 6.41%, PE and PVC by 2.56%, and PET by 3.85%. In contrast, the introduction of the same MPs into soils planted with *S. nigrum* resulted in a decrease in pH. The observed reductions were 6.10% for PP, 4.88% for PE and PVC, and 6.10% for PET. This contrast can be attributed to the divergent nature of the plants’ root exudates and their differential influence on soil processes [50]. In soils cultivated with *P. vittata*, the addition of MPs (PP, PE, PVC, and PET) may enhance microbial degradation of carboxylic acids, which consumes the hydrogen ions in the soil and consequently shifts the pH toward alkalinity [29]. Conversely, for *S. nigrum*, soil acidification induced by the same MPs may stem from increased root secretion of organic acids [51,52]. Therefore, the effect of MPs on soil pH fundamentally depends on the plant species and the specific biochemical processes that they induce.

**Figure 6:**
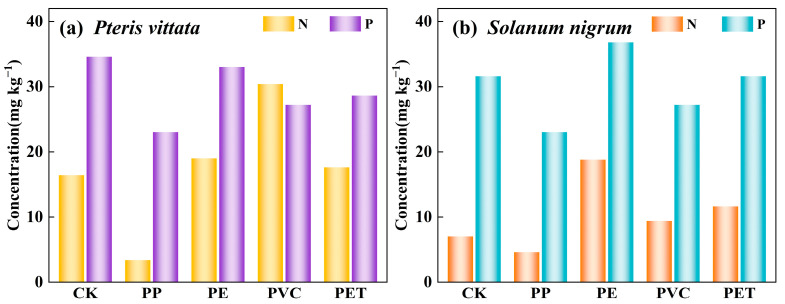
Effects of MPs on available N and P in soil of *Pteris vittata* (**a**) and *Solanum nigrum* (**b**). (N: nitrogen; P: phosphorus; CK: blank control group; PP: polypropylene; PE: polyethylene; PVC: polyvinyl chloride; PET: polyethylene terephthalate).

Figure 6 illustrates the alterations in the N and P contents within the soil cultivated with *P. vittata* and *S. nigrum* as induced by MP treatment. In soils supporting *P. vittata*, the N content ranged from 3.4 to 30.4 mg kg^−1^, while the P content fell between 23.0 and 34.6 mg kg^−1^ (Figure 6a). In comparison, soils planted with *S. nigrum* showed N levels of 4.6 to 18.8 mg kg^−1^ and P levels ranging from 23 to 36.8 mg kg^−1^ (Figure 6b). With the exception of PP, MPs such as PE, PVC, and PET all contributed to an increase in soil N concentrations. Su et al. [53] suggested that exposure to MPs enhanced denitrification in soil, leading to an increase in N_2_O emissions. Furthermore, pot experiments have shown that MPs can improve soil porosity, thereby enhance soil aeration and promote the release of N [54,55]. Our results indicated that the incorporation of PP significantly reduced soil N levels. This finding is consistent with the research conducted by Zhang et al. [56], suggesting a potential disruption of PP to soil nitrogen metabolism. Typically, MPs modify the availability of P by influencing soil ion exchange and adsorption [57]. In the present study, most MPs treatments led to a decrease in the soil P content, which may be attributed to the negatively charged surfaces of the MPs.

### 3.4. The Pollution and Potential Ecological Risk of Different Soil Types

Table 2 and Table 3 summarize the comprehensive assessment of Tl pollution levels and their associated potential ecological risks across different soil types, with a specific focus on the comparative analysis between soils without MPs intervention and those amended with MPs. In soils without MP addition (Table 2), the Tl pollution severity exhibited substantial variability among the tested soil types, reflecting inherent differences in soil geochemical properties and initial Tl accumulation.

Notably, the soil of Daliangzi exhibited an extremely high Tl pollution level, as indicated by the single-factor pollution index (*P_i_* = 7.99), a value that far exceeded the threshold for “very high contamination” (*P_i_* > 3) as per the standard assessment framework. Correspondingly, its potential ecological risk index (*E_r_* = 79.87) fell within the “moderate risk” category (*E_r_*: 40–80), signifying that Tl in this soil posed non-negligible threats to soil biota and surrounding ecosystems. In striking contrast, the soils cultivated with the two phytoremediation plants (*P. vittata* and *S. nigrum*) showed a marked attenuation of Tl pollution and ecological risks. Specifically, the *P_i_* values of Tl in the *P. vittata* and *S. nigrum*-planted soils decreased sharply from the “very high contamination” of the Daliangzi soil to the “moderate contamination” grade, with *P_i_* values recorded at 2.06 and 1.77, respectively. Concomitantly, their corresponding potential ecological risk indices (*E_r_*) were significantly reduced to the “low risk” level (*E_r_* < 40), with *E_r_* values of 20.56 (*P. vittata*) and 17.65 (*S. nigrum*). The significant reduction in the severity of pollution and ecological risk indicates the initial phytoremediation effect of *P. vittata* and *S. nigrum* on Tl pollution in soil without MPs. When MPs were present in the soil (Table 3), all *E_r_* values of Tl decreased markedly compared with those in MP-free soils (Table 2), with the ecological risk being classified as low.

## 4. Conclusions

This study investigated the impact of MPs and Tl on the bioaccumulation capacity of the hyperaccumulator plants *P. vittata* and *S. nigrum*, as well as their potential for remediating thallium-contaminated soil. Following treatment with *P. vittata* and *S. nigrum*, the *I_geo_* of Tl in the soil decreased to 0.454 and 0.235, respectively, corresponding to a pollution level classified as unpolluted to moderately polluted. Compared to the pre-plantation conditions, both plants significantly reduced the degree of Tl contamination in the soil, indicating that *P. vittata* and *S. nigrum* possess a strong capacity for Tl adsorption and remediation. PE exhibited markedly contrasting effects on Tl accumulation in *P. vittata* and *S. nigrum*. Specifically, PE promotes the uptake and accumulation of Tl in *P. vittata*, whereas under the same conditions, it inhibits Tl enrichment in *S. nigrum*. This discrepancy indicates that the interaction between PE and plants is species-specific, likely associated with differences in the plants’ inherent heavy-metal uptake mechanisms, their ability to modulate the rhizosphere microenvironment, and their physiological responses to MPs. MPs can alter the pH value of soil and the contents of N and P. According to the assessment of the potential ecological risk index, the Tl pollution in the Daliangzi Pb-Zn mine area has reached a very high contamination level, and the ecological risk is at a moderate risk level, reflecting that there is a significant Tl pollution problem in this area and posing a certain threat to local ecological security. In the soil planted with *P. vittata* and amended with PE, both the *P_i_* and *E_r_* reached the lowest values, recorded as low as 0.767 and 7.67, respectively. This indicates that PE can effectively enhance the adsorption and immobilization of Tl by *P. vittata*, thereby further improving its remediation efficiency in contaminated soil. However, in the soil cultivated with *S. nigrum*, it was PP that exhibited the lowest values.

## 5. Environmental Implications

From an environmental impact and management perspective, this finding is of critical importance. It indicates that in co-contaminated sites where MPs are widely present, traditional phytoremediation strategies must be optimized and upgraded. Future remediation practices should first identify the dominant types of MPs in the soil, and then precisely pair them with hyperaccumulator plants that exhibit positive synergistic effects, thereby maximizing remediation efficiency, shortening the treatment duration, and reducing secondary environmental risks. The study also confirmed that typical areas such as the Daliangzi mining region are under severe Tl contamination pressure, making the implementation of such precise and efficient remediation urgently necessary. In summary, incorporating MPs into the framework of risk assessment and remediation technology selection for contaminated sites is a scientific prerequisite for developing novel green remediation technologies tailored to real-world complex environments and for ensuring long-term ecosystem security.

## 6. Limitations

This study focused on the adsorption efficacy of *P. vittata* and *S. nigrum* for Tl at specific contamination levels, and it preliminarily examined the influence of microplastic addition on this process. However, the experiment did not investigate the absorption capacity limits of the two plants for Tl across different concentration gradients and under varying MPs conditions. Future work could incorporate additional experimental groups with varying concentrations to further explore synergistic remediation mechanisms for the co-contamination of Tl and Ms.

## Figures and Tables

**Table 1:** The multivariate geochemical evaluation of this study.

Equation No	Index	Principle	Explanation	Pollution Degree Classification
1.	Geo-accumulation Index (*I_geo_*)	*I_geo_* $=\log_{2}\left(\frac{\sigma }{1.5\times \varepsilon }\right)$	The σ represents the measured concentration (mg kg^−1^) of a specific HMs in the soil sample, 1.5 is a constant, and ε represents the corresponding background concentration (mg kg^−1^) of that HM in Sichuan Province soils. The values are Tl = 0.548, Ba = 474, Pb = 30.9, Cr = 79.0, and V = 96.0.	*I_geo_* ≤ 0, unpolluted; 0 < *I_geo_* ≤ 1, unpolluted to moderately polluted; 1 < *I_geo_* ≤ 2, moderately polluted; 2 < *I_geo_* ≤ 3, moderately to heavily polluted; 3 < *I_geo_* ≤ 4, heavily polluted; 4 < *I_geo_* ≤ 5, heavily to extremely polluted; *I_geo_* > 5, extremely polluted. [39]
2.	Bioconcentration Factor (*BCF*)	*BCF* $=\frac{C_{Plant}}{C_{Soil}}$	The $C_{Plant}$ represents the Tl content in the plant, and $C_{Soil}$ represents that in the soil.	/ [40]
3.	Single Factor Pollution Index (*P_i_*)	*P_i_* $=\frac{C_{i}}{S_{i}}$	The $C_{i}$ represents the measured content of Tl in the soil (mg kg^−1^), and $S_{i}$ represents the background value of Tl in Sichuan Province soils (Tl = 0.548 mg kg^−1^).	*P_i_* < 1, low contamination; 1 ≤ *P_i_* < 3, moderate contamination; 3 ≤ *P_i_* < 6, considerable contamination; *P_i_* ≥ 6, very high contamination. [41]
4.	Potential Ecological Risk Index (*E_r_*)	*E_r_* $=T_{r}\times P_{i}$	The $T_{r}$ represents the toxicity coefficient for Tl (Tl = 10).	*E_r_* ≤ 40, low risk; 40 < *E_r_* ≤ 80, moderate risk; 80 < *E_r_* ≤ 160, considerable risk; 160 < *E_r_* ≤ 320, high risk; *E_r_* > 320, Very high risk. [42]

**Table 2:** Assessment of Tl contamination and its associated risk in various soil types without MPs.

Soil Type	*P_i_*	Pollution Level	*E_r_*	Risk Level
Soil of Daliangzi	7.99	very high contamination	79.87	moderate risk
Soil of *P. vittata*	2.06	moderate contamination	20.56	low risk
Soil of *S. nigrum*	1.77	moderate contamination	17.65	low risk

*P_i_*: Single Factor Pollution Index; *E_r_*: Potential Eco-logical Risk Index; *P. vittata*: *Pteris vittata*; *S. nigrum*: *Solanum nigrum*.

**Table 3:** Assessment of Tl contamination and its associated risk in various soil types with MPs.

Soil Type	*P_i_*	Pollution Level	*E_r_*	Risk Level
Soil of *P. vittata* (PP)	0.835	low contamination	8.35	low risk
Soil of *P. vittata* (PE)	0.767	low contamination	7.67	low risk
Soil of *P. vittata* (PVC)	0.913	low contamination	9.13	low risk
Soil of *P. vittata* (PET)	1.049	moderate contamination	10.49	low risk
Soil of *S. nigrum* (PP)	0.779	low contamination	7.79	low risk
Soil of *S. nigrum* (PE)	0.862	low contamination	8.62	low risk
Soil of *S. nigrum* (PVC)	0.945	low contamination	9.45	low risk
Soil of *S. nigrum* (PET)	0.943	low contamination	9.43	low risk

*P_i_*: Single Factor Pollution Index; *E_r_*: Potential Eco-logical Risk Index; *P. vittata*: *Pteris vittata*; *S. nigrum*: *Solanum nigrum*; PP: polypropylene; PE: polyethylene; PVC: polyvinyl chloride; PET: polyethylene terephthalate.

## Data Availability

The original contributions presented in this study are included in the article. Further inquiries can be directed to the corresponding author.
